# Remarks on the Use of Vivisection, as a Means of Scientific Research

**Published:** 1845-01

**Authors:** 


ART. XII.—*Remarks on the Use of Vivisection, as a means of scientific research; in a Letter addressed to the Earl of Caernarvon, President of the Society for preventing Cruelty to Animals.* By RICHARD JAMESON.—*London*, 1844. 8vo, pp. 52.

IT appears that the society in question, a few years since, honoured with a prize of £100, an essay by the Rev. John Styles, D.D., on the claims of the animal creation on our humanity; in which essay, a multitude of most absurd fictions are propagated, in regard to the cruelties practised by medical men upon the unfortunate subjects of their experiments. Thus the reverend doctor gravely informs the public " that every surgeon's apprentice thinks himself entitled to find his way into the arcana of nature by scalping cats and rabbits to see where their brains lie ;" and much more to the same effect. We feel much inclined to believe with Mr. Jameson, that in his search after *horrors*, wherewith to gratify the taste of his judges, and render his arguments the more pointed, Dr. Styles encountered some waggish students who thought to satisfy his love of the marvellous by telling him horrible stories of " what they did at the hospital."

The positions which Mr. Jameson undertakes to prove, are the following:

"1st. That the *number* of experiments practised upon living animals is grossly exaggerated by the writers employed by the society.

"2d. That the *pain* caused by these experiments is also in many cases exaggerated.

"3d. Admitting that, in many instances, much pain is the result, vivisection is notwithstanding *necessary* as a means of acquiring and of imparting useful knowledge.

"4th. But whilst necessity alone compels us to take away the lives of animals, *our accusers* are daily accessory to their wholesale destruction and torture, for the mere gratification of luxury, or as an amusement to while away the passing hour."

In these positions we entirely accord with the author, and we think that he has sustained them well. Our readers must be well acquainted with our own views on the subject, which are briefly these: that, to establish an important position, or to resolve a doubtful question, whether its consequences have an immediate practical bearing, or are *at present* interesting merely in a scientific point of view, (all science must *ultimately* have a beneficial influence on our practice,) vivisection is justifiable, but that recourse should not be had to this means of investigation, until all possible light has been obtained from other sources of inquiry; that the experiments should be so devised as to cause the smallest amount of animal suffering, and that when their end is once answered, they should not be needlessly repeated.

We cannot think that the British school of physiology is chargeable with much error in this respect; whatever may be the faults of our continental brethren.

